# Five-Year Enhanced Natural Attenuation of Historically Coal-Tar-Contaminated Soil: Analysis of Polycyclic Aromatic Hydrocarbon and Phenol Contents

**DOI:** 10.3390/ijerph18052265

**Published:** 2021-02-25

**Authors:** Arkadiusz Telesiński, Anna Kiepas-Kokot

**Affiliations:** 1 Department of Bioengineering, Faculty of Environmental Management and Agriculture, West Pomeranian University of Technology in Szczecin, Słowackiego Str. 17, 71-434 Szczecin, Poland; 2 Department of Environmental Management, Faculty of Environmental Management and Agriculture, West Pomeranian University of Technology in Szczecin, Słowackiego Str. 17, 71-434 Szczecin, Poland; anna.kiepas-kokot@zut.edu.pl

**Keywords:** naphthalene, PAHs, phenols, remediation, soil

## Abstract

The objective of this study was to assess the soil pollution on an industrial wasteland, where coal-tar was processed in the period between 1880 and 1997, and subsequent to assess the decline in the content of phenols and polycyclic aromatic hydrocarbons (PAHs) during enhanced natural attenuation. The soil of the investigated area was formed from a layer of uncompacted fill. Twelve sampling points were established in the investigated area for collecting soil samples. A study conducted in 2015 did not reveal any increase in the content of heavy metals, monoaromatic hydrocarbons (BTEX), and cyanides. However, the content of PAHs and phenols was higher than the content permitted by Polish norms in force until 2016. In the case of PAHs, it was observed for individual compounds and their total contents. Among the various methods, enhanced natural attenuation was chosen for the remediation of investigated area. Repeated analyses of the contents of phenols and PAHs were conducted in 2020. The results of the analyses showed that enhanced natural attenuation has led to efficient degradation of the simplest substances—phenol and naphthalene. The content of these compounds in 2020 was not elevated compared to the standards for industrial wastelands. The three- and four-ring hydrocarbons were degraded at a lower intensity. Based on the mean decrease in content after 5-year enhanced natural attenuation, the compounds can be arranged in the following order: phenols > naphthalene > phenanthrene > fluoranthene > benzo(a)anthracene > chrysene > anthracene.

## 1. Introduction

Soil pollution resulting from human activity poses a significant threat to the given ecosystem [1]. One of the byproducts of the coking process is coal-tar [2], which is a mixture of organic compounds of variable composition. It is formed primarily from aromatic hydrocarbons. Among the hydrocarbons, naphthalene is the highest contributor by weight (approximately 10%), followed by benzo(a)anthracene, benzo(b,k)fluoranthene, dibenzo(a,h)anthracene, chrysene, benzo(a)pyrene, benzene, and phenolic compounds [3]. Coal-tar is a chemically stable substance exhibiting low reactivity. In ambient temperature conditions, the evaporation rate of its ingredients is low and increases with temperature [4].

The bioavailability of pollutants is primarily determined by their physicochemical properties and the medium in which they are found [5]. Depending on the complexity of systems, such as soil, organic pollutants can occur both in an adsorbed form (on soil particles) and in a free form (in soil air and in soil water) [6].

Due to the ongoing devastation and pollution of the environment with hydrocarbons and phenols, there is a need for intensive treatments aiming at the restoration of the natural status of the soil [7]. Depending on the ecosystem in which they are found, pollutants may undergo slow transformations resulting from various chemical, physical, and biological processes. Highly toxic soil pollution may result in the disappearance of organisms, thus disabling the self-purification process of the soil environment [8].

Polycyclic aromatic hydrocarbons (PAHs) are chemicals containing two or more planar condensed aromatic rings. About 100 homocyclic hydrocarbons have been discovered in the environment, in addition to several hundreds of their alkyl-, amine-, and nitro-derivatives. Furthermore, heterocyclic PAHs are found, containing of oxygen, sulphur, or nitrogen atoms [9].

The aromatic rings of PAHs are arranged in a linear, angular, or cluster manner, and contain greater or lower amounts of alkyl or nitro-substituents [5]. PAHs are characterized by strong hydrophobic and lipophilic properties, low water solubility, low volatility, and strong sorption affinity toward the soil organic matter [10,11,12]. The majority of PAHs exhibit relatively high stability in the environment. This is primarily due to the exceptionally strong bonds in their system of condensed aromatic rings, observed in the form of a cloud of delocalized π electrons, which also contributes to their characteristics, such as a low ratio of hydrogen to carbon atoms, relatively strong C–C bond, tendency for substitution reactions, and electron delocalization within carbon atoms (resonance stabilization) [13]. PAHs have different structures, in which the benzene rings assume various orientations relative to each other. In certain PAH molecules, characteristic areas referred to as the K region (external border of the phenanthrene ring) and M region (opposing atoms of the anthracene structure) are observed. In addition, the location of another region known as the bay region is considered important. These are the areas and positions attributed by researchers to the biological activity of PAHs [14].

PAHs are often accompanied by other pollutants, namely phenolic compounds [15]. Phenol and its derivatives are known as a common component of the soil polluted by different industries, including crude oil refineries, oil industry, textile industry, or coal refineries [16]. The toxicity of phenol has been widely documented, and its overkill impact on humans and the environment was reported as highly concerning.

It is generally assumed that soils exposed to hydrocarbons in the past often pose challenges for the remediation process. Removal of crude oil derivatives is mostly delayed by the absence of microorganisms that are capable of degradation, by an insufficient supply of nutrients, in order to balance the use of carbon associated with such residues, and finally by the lack of access of microorganisms to these residues of oil derivatives, which prevents their biodegradation [17]. Our study was conducted with an aim of assessing the self-purification intensity of phenols- and PAHs-contaminated soil from an area where a coal-tar processing plant was formerly present.

## 2. Materials and Methods

### 2.1. Study Area Characteristics

**Figure 1:**
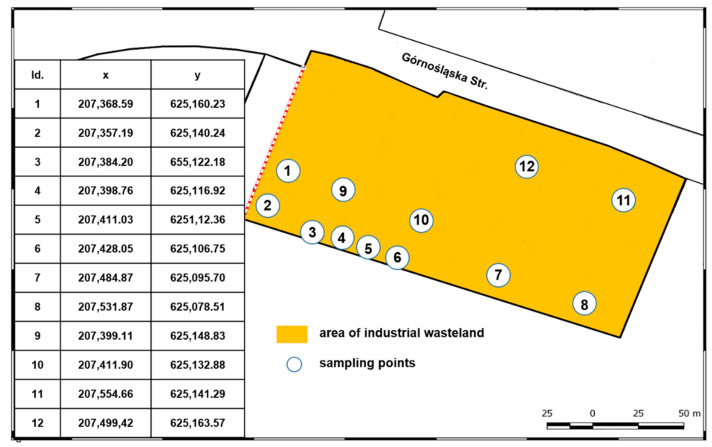
Location of sampling points on an industrial wasteland with historically coal-tar-contaminated soil.

The study was carried out on an area of approximately 2.5 ha in Szczecin, Poland, after ca. 20 years from the liquidation of the last operated industrial system that processed coal-tar into insulation roofing felt. Industrial activity had been carried out in the area continuously from approximately 1880 to 1997, with changing ownership and production technology. Currently, the area is an industrial wasteland (Figure 1).

As the same activity was carried out on the discussed area for a long term, it was highly likely that over 30 years had passed from soil contamination, and thus the pollution was classified as historical. The roofing felt production system was disassembled 2–3 years prior to the official liquidation of the last plant. Considering the low standards of waste management in 1970s, it could be assumed that a considerable portion of the pollution emission to the soil occurred in this period due to improper practices of material and production waste management. The pollution emission resulted from the activity rather than an event, and thus it remains difficult to determine the date of introduction of pollutants to the soil. Before the remediation activities were commenced in 2015, all buildings, structures, and waste were removed from the site.

### 2.2. Soil Properties and Type of Area Coverage, Including Vegetation and Development

The soil on the investigated area was formed from a layer of uncompacted fill, which was introduced to improve the load-bearing conditions of the ground for laying the foundation of buildings and structures. The thickness of the fill layer ranged between 1.8 and 2.8 m. The fill was executed on the organic horizon comprising aggregate mud and peat with a high thickness (approximately 10 m). The upper layer of the fill consisted primarily of sand. At a depth of about 0.5 m below the fill layer, the proportion of sand and loam-sand material was increased.

The study area is characterized by a low degree of permanent soil coverage with technical infrastructure (approximately 10%), with a high degree of decapitalization, constituting surface hardening. A high contribution of the bioactive surface is found in this area, which is not covered with permanent pavement (approximately 90%), and is subject to natural succession of turf vegetation, as well as shrubs and trees. No potential sources of hydrocarbon pollution (such as reservoirs and installations) are currently present in the polluted area. The persisting coverage of the area with vegetation (apart from where vegetation has been mechanically destroyed) indicates the lack of toxic effects toward plants (the soil retained the vegetation conditions despite the pollution).

### 2.3. Determination of the Content of Soil Pollutants

Soil pollution testing was conducted following the legal regulations applicable in Poland at the time of analyses, which did not present any reference methodologies for the testing of soil quality. Therefore, the general principles for performing them in accredited laboratories with the use of accredited methods were applicable.

Testing for the confirmation of soil pollution was carried out in September 2015 through the analysis of samples collected from 12 sites on the investigated industrial wasteland. Analyses of pollutant contents were performed at depths between 0 and 2 m (with the following divisions: 0–0.25, 0.25–1.0, and 1.0–2.0 m). Based on the specificity of the production activity conducted on the investigated area in the past, a list of substances that were expected to occur was drawn up: PAHs (naphthalene, phenanthrene, anthracene, fluoranthene, chrysene, benzo(a)anthracene, benzo(a)pyrene, benzo(a)fluoranthene, benzo(ghi)perylene, total PAHs), metals (As, Cr, Zn, Cu, Ni, Pb, Hg), BTEX (benzene, toluene, ethylbenzene, xylene; as well as styrene and total BTEX), cyanides (free and bound), and phenolic index.

To determine the efficiency of enhanced natural attenuation, further soil testing was carried out in July 2020. This included the analyses of pollutant contents, which exceeded the permissible standards in Poland in 2015.

Analyses of the content of monoaromatic hydrocarbons, PAHs, and cyanides were conducted by AB 918 laboratory. Among the protocols used for testing, the content of monoaromatic hydrocarbons was analyzed using gas chromatography with flame-ionization detection (GC-FID) in accordance with PN-ISO 22155:2013 [18], and the content of cyanides using a spectrophotometric method according to PN-ISO 11262:2008 [19]. PAH content was determined by gas chromatography with flame-ionization detection (GC-FID), according to the accreditation of the laboratory that performed the analysis.

Analyses of the phenolic index and content of metals were carried out by AB 868 laboratory. The protocols used for testing the phenolic index of soil involved the application of the spectrophotometric method with 4-aminoantipyrine after distillation [20]. The determination of cadmium, chromium, copper, lead, nickel, and zinc was carried out with atomic absorption spectrometry [21]. On the other hand, the laboratory stated that the PB-38/PS procedure of 1 March 2011 was used for mercury determination, which indicates the application of updated methods described in the standards/procedures developed by the laboratory.

The content of the determined soil pollutants was expressed as the weighted average:

(1) $$\bar{x}=\frac{\sum_{i=1}^{n}x_{i}w_{i}}{\sum_{i=1}^{n}w_{i}}$$

where $x_{i}$ is the pollutant content at the given depth and $w_{i}$ is the thickness of the determined horizon.

### 2.4. Soil Remediation Methods

A total of 27 parameters were investigated, which included the determination of excessive levels of phenols and six PAHs. Exceedance of the permissible content in the studied soil was also determined for total PAHs.

Remediation activities were commenced on the analyzed industrial wasteland, which were established with the corresponding environment conservation body. Three remediation methods were considered: enhanced natural attenuation, soil flushing, and soil replacement. Cost-benefit analysis was carried out for all three remediation variants.

The conducted analysis revealed that a relatively high number of technical solutions exist for the investigated case, enabling the removal of pollution and to revive the soil to the state required by norms. Each of the analyzed method made it possible to achieve the assumed effect. The only differences in the variants concerned the real costs necessary to obtain the expected effect and the external (environmental) costs resulting from the applied solutions.

## 3. Results

### 3.1. The Content of Pollutants in Soil on the Area of the Industrial Wasteland Prior to the Remediation Commencement

The occurrence of heavy metals, BTEX and cyanides in soils in amounts higher than the Polish limit values for industrial areas was not confirmed in the studied area. However, exceedances of soil quality norms applicable until 2016 were observed for PAHs and phenols. In the case of PAHs, exceedances of norms were observed for both individual compounds and their total amount. Exceedances of the permissible content of total PAHs were determined in 11 and phenols in six out of 12 tested sites (Table 1).

### 3.2. Selection of Soil Remediation Method

The conducted analysis showed that enhanced natural attenuation will be the most rational solution for the analyzed soil, considering the performance of the necessary agrotechnical earthworks, maintenance of the vegetation cover, monitoring and testing. It was furthermore established that if the rate and effect of remediation are unsatisfactory, it will be possible to introduce supportive actions for enhanced natural attenuation.

A series of arguments supported the use of enhanced natural attenuation on the tested industrial wasteland. Soil pollution in this area resulted from the excessive presence of organic compounds with different degrees of susceptibility to biodegradation, and the exceedance of the permissible content of the substances posing risk was not considerable. The content of hydrocarbons was dominated by those compounds with relatively good susceptibility to biological degradation. The long-term exposure of soil to contamination with organic compounds (carbon derivatives) on the pollution site favored the selection and adaptation of microorganisms to the prevailing conditions, which had a positive effect on the autochtonous biodegradation potential. The dispersion of pollutants within a large area hindered the identification of areas with greater pollution levels (foci), which could be cleaned with ex situ methods using rational expenditure levels. The land development status implied the use of in situ methods, which would not require soil movement or installation of remediation instrumentation.

The remediation works were commenced in January 2016. The remediation time was determined by the following variables: (i) content of total PAHs and phenols in the soils of an industrial wasteland which is permissible by Polish law (250 and 50 mg kg^−1^ DM, respectively); (ii) necessary reduction in the content of PAHs and phenols in the soils of an industrial wasteland, to the permissible level in industrially used soils; and (iii) rate of degradation of phenols and PAHs in soil.

Boopathy [22] stated that the rate of hydrocarbon degradation in the surface layers of soils ranges between 2 and 40 mg kg^−1^ soil d^−1^, with an average of 9–14 mg kg^−1^ soil d^−1^. In deeper layers of soil, the degradation rate decreases due to the lower oxygen concentration and microorganism counts. However, these indices can be achieved with the so-called fresh pollution, with a high contribution of hydrocarbon volatilization to the atmosphere and stimulation of biodegradation processes by creating suitable degradation conditions [23].

The analyzed case was characterized by historical pollution. In such conditions and assuming that the process of hydrocarbon reduction will result from self-purification, the expected reduction rate will be below 2 mg kg^−1^ soil d^−1^. Such forecasts of phenol and PAH kinetics resulted from the following conditions: (i) characteristics of the so-called old pollution, where the reduction due to volatilization to the atmosphere is low; (ii) occurrence of a mixture of four-ring PAHs at an exceeded permissible content on the analyzed area (fluoranthene, chrysene, benzo(a)anthracene), which are more difficult to decompose due to hydrocarbons with lower number of rings; and (iii) high spatial variability of the PAH content in the soil (min. 174 mg kg^−1^ DM, max. 1235 mg kg^−1^ DM) (Table 1).

Given the maximum content of PAHs (mg kg^−1^ DM) and the assumption of reduction below 2 mg kg^−1^ soil d^−1^, it was suggested that the remediation process to reach the required amount should take approximately 3–4 years.

### 3.3. Phenol Content in Soil after Enhanced Natural Attenuation

**Figure 2:**
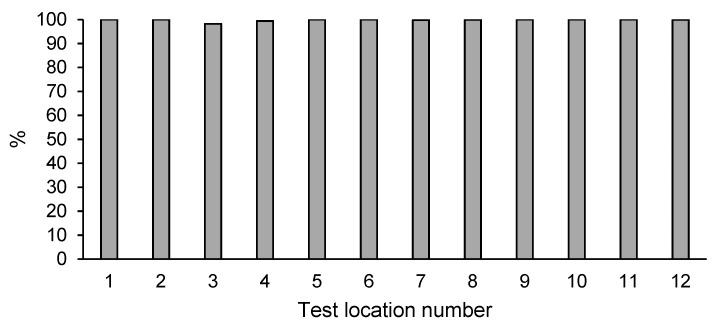
Percentage decrease of phenol content in the soil after enhanced natural attenuation.

None of the examined points (1–12) had excessive content of phenols (50 mg kg^−1^ DM) (Table 2). The phenol content in the samples examined prior to and after remediation was found to be significantly reduced by 98–100% (Figure 2). The concentration in the samples with an initial phenol content not exceeding 20 mg kg^−1^ DM, as well as those containing over 100 mg kg^−1^ DM, did not exceed 4 mg kg^−1^ DM in 2020. In a large portion of samples, the determined phenol content was lower than the quantification level. The obtained reduction of phenol was in line with the expectations.

### 3.4. Naphthalene Content in Soil after Enhanced Natural Attenuation

**Figure 3:**
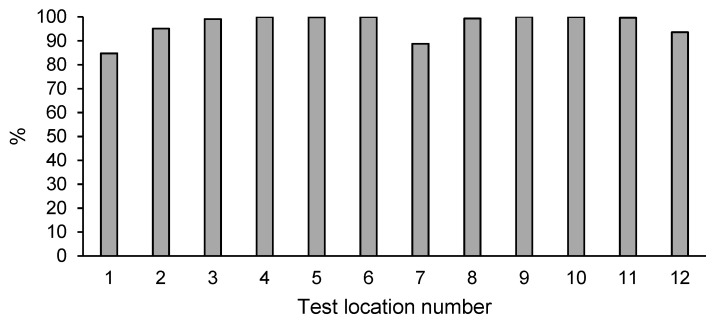
Percentage decrease of naphthalene content in the soil after enhanced natural attenuation.

The high efficiency of naphthalene degradation was confirmed in all test points (1–12) (Table 3). The percentage of naphthalene removal during enhanced natural attenuation ranged from 85% to 100% (Figure 3). The obtained naphthalene reduction was consistent with the remediation assumptions. Naphthalene, which is the simplest PAH with two condensed benzene rings, is the most easily decomposable and relatively volatile compound of the PAH group. Although the initial levels of naphthalene on the investigated area were very high, the effect of its degradation was highly satisfactory and a significant reduction effect was observed in the entire area. In light of the obtained results, the tested area was qualified as free of naphthalene pollution.

### 3.5. Three-Ring PAH Content in Soil after Enhanced Natural Attenuation

The majority of the test points were characterized by soil, in which the content of three-ring PAHs was below the level expected to be achieved with remediation (Table 4). None of the samples revealed excessive content of phenanthrene or anthracene. It shall be considered that the content of three-ring PAHs in the area demarcated by these test points (1–6, 9–11) was brought to the level required by the decision establishing the remediation plan.

However, in soil samples collected from points 7, 8, and 12, the content of three-ring PAHs exceeded the levels set out in Polish soil quality standards applicable until 2016. Furthermore, in comparison with 2015, an increase in the content of phenanthrene was found in 2020 at point 7 and an increase in anthracene content at points 7, 8, and 12.

### 3.6. Four-Ring PAH Content in Soil after Enhanced Natural Attenuation

Similar to naphthalene and three-ring hydrocarbons, the majority of test points were characterized by a lower content of four-ring PAHs (covered by the remediation plan) compared to the level required to be obtained after remediation (Table 5). None of the samples revealed excessive content of fluoranthene, chrysene, and benzo(a)anthracene. It shall be considered that the content of four-ring PAHs in the area demarcated by these test points (1–6, 9–11) was brought to the level required by the decision establishing the remediation plan.

However, in the soil samples collected in point 7, the content of four-ring PAHs exceeded the levels set out in the Polish soil quality standards applicable until 2016, whereas in points 8 and 12 it was observed only for the content of fluoranthene. Furthermore, in addition, compared to 2015, in 2020 an increase in chrysene and benzo(a)anthracene contents was reported in point 7.

### 3.7. Total PAH Content in Soil after Enhanced Natural Attenuation

**Figure 4:**
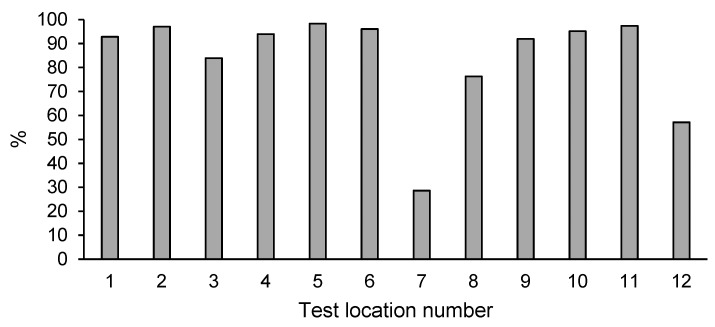
Percentage decrease of total PAH content in the soil after enhanced natural attenuation.

All the test points (1–12) were found to have decreased total PAHs content after enhanced natural attenuation (Table 6). The content in 2020 was 29–98% lower than the level determined in 2015 (Figure 4). With the exception of points 7 and 12, no excessive total PAH concentration could be found in the studied area. Therefore, it can be assumed that the total PAH content in the majority of the analyzed area was brought to the level required by the decision setting out the remediation plan.

### 3.8. Mean Decrease of Phenol and PAH Content in Soil after Enhanced Natural Attenuation

**Figure 5:**
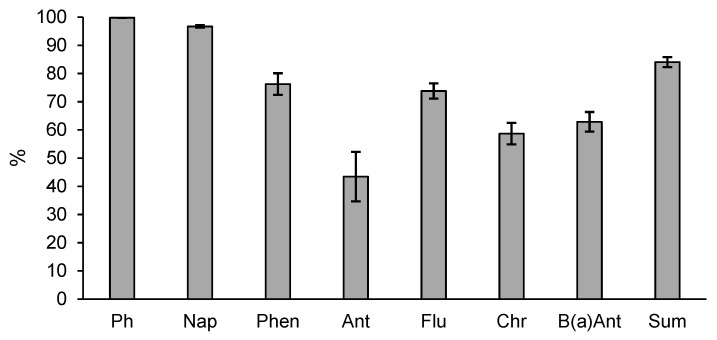
Mean percentage decrease of phenols and PAHs in the soil after enhanced natural attenuation; data are expressed as mean ± standard error; Ph—phenols, Nap—naphthalene, Phen—phenanthrene, Ant—anthracene, Flu—fluoranthene, Chr—chrysene, B(a)Ant—benzo(a)anthracene, Sum—sum of PAHs.

A comparison of the mean percentage reduction in the content of phenols and PAHs in the soil after enhanced natural attenuation showed the highest decrease for phenols (99%) and for naphthalene (97%). The lowest mean decrease was determined for anthracene (43%). The pollutants, whose concentration prior to the commencement of enhanced natural attenuation in at least one point exceeded the Polish permissible soil quality standards, can be arranged in the following order based on the mean content decrease in the content: phenols > naphthalene > phenanthrene > fluoranthene > benzo(a)anthracene > chrysene > anthracene. The total PAH content was reduced during enhanced natural attenuation by 84% (Figure 5).

## 4. Discussion

Due to their toxic, mutagenic, and carcinogenic properties, pollution of soil with phenols and PAHs may pose a hazard to all living organisms of the given ecosystem [24]. The reaction of both soil microorganisms and plants to the pollutants depends on the environmental and soil factors and the properties of chemical compounds, as well as their time of contact (for a xenobiotic). The problem is markedly aggravated in the case of historical pollution, due to the slow sorption, diffusion, or equilibrium division of pollutants throughout the years [25]. Another important issue is pollution aging. Two possible explanations can be given for this phenomenon in soils: organic matter diffusion and sorption-retarded diffusion [26].

This study was conducted in an industrial wasteland, where coal-tar was processed into insulation roofing felt until 1997. Coal-tar is formed as a byproduct of the coking process, in a liquid form as a dense, black fluid. Considering the fact the investigated area was characterized by historical soil contamination with coal-tar, the substance can be practically absent in its original (liquid) form, but bound with the soil and ground structure (adsorbed on soil particles), forming impregnation lumps in some areas.

The study conducted in 2015 did not reveal an excessive content of heavy metals, BTEX, and cyanides in any of the 12 sampling sites. However, exceedances of Polish soil quality standards (applicable until 2016) were determined for PAHs and phenols. Numerous authors have pointed to the increased concentrations of phenol and PAHs in soil on the areas of coal-tar processing plants [27,28,29,30]. Creosote is an additional product of coal-tar distillation. As stated by Smułek et al. [31], the industry that used coal-tar or creosote contributed to approximately 4% of all soil pollution in Europe, while PAHs constituted almost 11%. It has been shown that in the areas of plants where worn railway ties impregnated with creosote were stored or reused, the content of PAHs in soil exceeded the applicable standards [15,32,33].

Due to the exceedance of soil quality standards in relation to the content of phenols and PAHs, remediation works were started in the given industrial wasteland. The conducted analysis showed that the most rational solution for the remediation of the investigated area will be enhanced natural attenuation. Considering that the area was characterized by historical contamination, it was highly likely that specific autochtonous strains supporting the biodegradation of the present pollution were developed [24]. The ongoing process of self-purification pointed to the considerable degradation potential of the soil, which in the period of several years could contribute to reviving it to the state required by the standards.

The process of enhanced natural attenuation conducted for a period of five years has led to efficient degradation of the simplest hydrocarbons—phenols and naphthalene—covered by the decision setting out the remediation plan. The obtained results are in line with the data provided by other authors [15,34,35,36,37,38,39].

Phenol, which is the main ingredient of coal-tar (the source of pollution of the investigated area), is the simplest representative of single-ring aromatic hydrocarbons in terms of the chemical structure [40]. It also has the simplest structure among the pollutants tested in the discussed area. This facilitates its breakdown (particularly under aerobic conditions and in the presence of microorganisms), the efficiency of which has been confirmed by research [41].

Phenolic compounds in soil may be degraded under both aerobic and anaerobic conditions [42]. Some microorganisms that can decompose these compounds are metabolically versatile, i.e., capable of degrading, apart from phenol, a wide range of substrates, such as naphthalene. These organisms include, but are not limited to, bacteria such as *Pseudomonas* sp., *Bacillus* sp., *Xanthomonas* sp., *Rhodococcus* sp., and *Sphingomonas* sp. [43].

Furthermore, ligninolytic and nonligninolytic fungi contribute to the oxidation of aromatic rings to arene oxides catalyzed by monooxygenases. The enzymes of ligninolytic fungi have the capacity to mineralize aromatic rings unlike nonligninolytic fungi, which are capable of only partially degrading PAHs. In addition, ligninolytic fungi contain extracellular enzymes that can act on a wide range of aromatic compounds [44].

Degradation of compounds with an aromatic structure is associated with the activity of genes (e.g., catA, ndoB, todC) encoding the enzymes responsible for the hydroxylation and splitting of the ring into the intermediates of central metabolic pathways, as well as CO_2_ and H_2_O [45]. Aromatic compounds undergo biodegradation to one of the five basic products: catechol, protocatechuic acid, homocatechuic acid, homogentisic acid, or unconventional compounds such as salicylic acid, anthranilic acid, and orthoaminophenol. Finally, during the biodegradation of aromatic compounds, intermediates of the tricarboxylic acid cycle are formed, including acetylo-CoA, pyruvate, and succinate [44,45,46,47,48].

Aromatic compounds with the simplest structure and lower molecular weight are the most susceptible to degradation. This has also been confirmed by the results of the present study. The mean content of phenols and naphthalene was reduced during enhanced natural attenuation by 99.70% and 96.67%, respectively. An increase in the number of rings in PAHs results in their reduced susceptibility to biodegradation [23]. Anthracene was found to have the highest resistance to degradation among the tested compounds. It is also interesting that after enhanced natural attenuation, increased contents of mainly three-ring PAHs (phenanthrene and anthracene) were observed in certain points. As presented by Boulangé et al. [30], such results may be seen in areas with a high concentration of pollutants due to a prolonged industrial activity carried out on the soil surface, as in the presented case. Thus, the formed local foci of pollutants result in a mosaic pattern. Furthermore, repeated sample collection on the same sites results in local contamination movements, and thus, the variability caused by distribution may not be captured [49]. In the case of simple, readily degradable hydrocarbons (phenol, naphthalene), the high degradation dynamics dominated over the variability caused by their distribution in soil.

The increased content of certain PAHs may also be related to their desorption from the organic matter and loamy fraction of the soil [50]. However, according to Barnier et al. [51], old-PAH-contaminated soils have a very small rapidly desorbing fraction. Richardson and Aitken [52] stated that desorption of PAHs in soils typically has two phases—the initial stage of rapid release followed by a longer period of slow desorption of these compounds. However, Birdwell and Thibodeaux [53] indicated that some soils show a transition from slow- to fast-desorbing domains. Moreover, Harmsen et al. [23] determined three stages of biodegradation in the soil for PAHs: rapid degradation within the first year, slow degradation in the next 6 years, and very slow degradation after 7 years from soil contamination. The authors also determined that during the very slow degradation stage, the rate of degradation of two-, three-, and four-ring PAHs was equal to that of five- and six-ring PAHs. However, the present study showed that in terms of the mean decrease of content during 5-year enhanced natural attenuation, the determined PAHs can be ordered as follows: naphthalene > phenanthrene > fluoranthene > benzo(a)anthracene > chrysene > anthracene.

## 5. Conclusions

The conducted study showed that five-year enhanced natural attenuation in the area with historical contamination with coal-tar has led to efficient degradation of the simplest pollutants determined in the soil—phenols and naphthalene. PAHs with a more complex structure (phenanthrene, anthracene, fluoranthene, chrysene, benzo(a)anthracene) were degraded at a lower efficiency. The results showed that a portion of the studied area has been brought to the state defined by the Polish standard of soil quality for industrial areas, and for which remediation works are no longer required. However, the remaining portion of the area would require a multiannual remediation process through enhanced natural attenuation in order to achieve a similar effect. Intensification of this process, with a chance of a satisfactory result over the next several years, would be linked to ecological imbalance in order to reach the contaminated layers and to stimulate the degradation of PAHs. Such intervention may, however, be a hazard rather than a benefit to the environment.

## Figures and Tables

**Table 1:** The lowest and highest contents of pollutants determined in the soil from the industrial wasteland prior to remediation, based on the permissible Polish standards for industrial areas (mg kg^−1^ DM).

Substance	Minimum Value	Maximum Value	Limit Value	Number of Points with Exceeded Limit Value
Metals				
As	n/a	6.00	60	0
Cr	2.60	8.98	500	0
Zn	70.4	102	1000	0
Cd	n/a	0.43	15	0
Cu	9.84	28.6	600	0
Ni	5.42	13.9	300	0
Pb	36.8	137	600	0
Hg	0.13	4.30	30	0
Monoaromatic hydrocarbons (BTEX)				
Benzene	0.02	1.80	100	0
Toluene	0.06	2.26	200	0
Ethylbenzene	0.02	1.41	200	0
Xylenes	0.26	5.70	100	0
Styrene	0.04	0.40	60	0
Sum of BTEX	0.40	11.4	200	0
Polycyclic aromatic hydrocarbons (PAHs)				
Naphthalene	1.51	266	50	9
Phenanthrene	8.44	229	50	10
Anthracene	6.96	116	50	3
Fluoranthene	26.7	190	50	6
Chrysene	8.79	100	50	3
Benzo(a)anthracene	8.18	134	50	5
Benzo(a)pyrene	6.91	35.0	50	0
Benzo(a)fluoranthene	2.14	9.79	50	0
Benzo(g,h,i)perylene	2.95	12.8	50	0
Sum of PAHs	174	1235	250	11
Others				
Free cyanides	n/a	n/a	40	0
Complex cyanides	0.02	0.23	40	0
Phenols	11.2	188	50	6

n/a—values below the detection level; BTEX—monoaromatic hydrocarbons; PAH—polycyclic aromatic hydrocarbons

**Table 2:** Changes in phenol content (mg kg^−1^ DM) in the soil prior to (2015) and after the completion of enhanced natural attenuation (2020).

Test Location Number	Content in 2015	Content in 2020
1	20.0	n/a
2	16.9	n/a
3	13.7	0.25
4	11.2	0.07
5	14.4	n/a
6	13.0	n/a
7	**138**	0.22
8	**132**	0.08
9	**103**	n/a
10	**147**	n/a
11	**169**	n/a
12	**188**	0.17

Results are expressed as the weighted average of concentration in the soil from the depth range of 0–2 m; bold font is used to mark the contents exceeding the Polish permissible soil norms; n/a—values below the detection level (0.05 mg kg^−1^ DM).

**Table 3:** Changes in naphthalene content (mg kg^−1^ DM) in the soil prior to (2015) and after the completion of enhanced natural attenuation (2020).

Test Location Number	Content in 2015	Content in 2020
1	1.53	0.23
2	36.7	1.79
3	**132**	1.22
4	**145**	0.04
5	**152**	0.18
6	**170**	0.08
7	**71.3**	7.99
8	**222**	1.46
9	5.52	n/a
10	**74.9**	0.07
11	**261**	0.95
12	**266**	17.0

Results are expressed as the weighted average of concentration in the soil from the depth range of 0–2 m; bold font is used to mark the contents exceeding the Polish permissible soil norms; n/a—values below the detection level (0.01 mg kg^−1^ DM).

**Table 4:** Changes in phenanthrene and anthracene content (mg kg^−1^ DM) in the soil before (2015) and after enhanced natural attenuation (2020).

Test Location Number	Phenanthrene		Anthracene	
	Content in 2015	Content in 2020	Content in 2015	Content in 2020
1	8.44	0.70	6.96	0.15
2	25.1	0.72	18.0	0.09
3	**70.3**	5.47	**116**	6.12
4	42.1	1.66	18.4	0.58
5	**85.7**	0.53	16.1	0.11
6	**79.0**	2.27	27.3	0.59
7	**160**	**232**	43.9	**133**
8	**143**	**23.1**	28.9	**67.5**
9	**73.1**	0.80	22.2	0.07
10	**143**	1.01	32.7	0.13
11	**183**	4.92	**65.1**	6.54
12	**229**	**212**	**93.4**	**110**

Results are expressed as the weighted average of concentration in soil from the depth range of 0–2 m; bold font is used to mark the contents exceeding the Polish permissible soil norms.

**Table 5:** Changes in the content of fluoranthene, chrysene, and benzo(a)anthracene (mg kg^−1^ DM) in the soil before (2015) and after enhanced natural attenuation (2020).

Test Location Number	Fluoranthene		Chrysene		Benzo(a)anthracene	
	Content in 2015	Content in 2020	Content in 2015	Content in 2020	Content in 2015	Content in 2020
1	31.8	1.77	13.8	2.26	10.4	1.98
2	28.4	1.46	12.3	0.96	9.26	0.77
3	28.2	8.39	9.59	9.62	8.67	9.94
4	26.7	3.93	10.1	4.55	9.24	3.48
5	32.0	1.54	8.79	1.19	8.18	1.10
6	37.0	4.64	10.5	2.55	10.5	2.31
7	**114**	**105**	47.7	**75.6**	**57.2**	**75.5**
8	**111**	**73.5**	40.1	24.4	**51.4**	21.5
9	**108**	0.32	35.9	7.41	34.4	6.38
10	**104**	0.92	**55.0**	6.81	**54.9**	6.56
11	**134**	9.62	**93.7**	8.62	**91.7**	4.87
12	**190**	**142**	**100**	27.3	**135**	28.2

Results are expressed as the weighted average of concentration in the soil from the depth range of 0–2 m; bold font is used to mark the contents exceeding the Polish permissible soil norms.

**Table 6:** Changes in total PAH content (mg kg^−1^ DM) in the soil before (2015) and after enhanced natural attenuation (2020).

Test Location Number	Content in 2015	Content in 2020
1	174	12.5
2	**257**	7.45
3	**527**	85.1
4	**468**	28.5
5	**491**	8.46
6	**503**	19.8
7	**903**	**645**
8	**897**	213
9	**501**	40.4
10	**915**	44.3
11	**1819**	47.4
12	**1235**	**530**

Results are expressed as the weighted average of concentration in the soil from the depth range of 0–2 m; bold font is used to mark the contents exceeding the Polish permissible soil norms.

## Data Availability

The data presented in this study are available on request from the corresponding author
